# Towards high-energy, high-resolution computed tomography via a laser driven micro-spot gamma-ray source

**DOI:** 10.1038/s41598-018-33844-7

**Published:** 2018-10-26

**Authors:** Y. C. Wu, B. Zhu, G. Li, X. H. Zhang, M. H. Yu, K. G. Dong, T. K. Zhang, Y. Yang, B. Bi, J. Yang, Y. H. Yan, F. Tan, W. Fan, F. Lu, S. Y. Wang, Z. Q. Zhao, W. M. Zhou, L. F. Cao, Y. Q. Gu

**Affiliations:** 1 Science and Technology on Plasma Physics Laboratory, Research Center of Laser Fusion, CAEP, Mianyang, Sichuan 621900 China; 2 IFSA Collaborative Innovation Center, Shanghai Jiao Tong University, Shanghai, 200240 China; 3 Department of Engineering Physics, Tsinghua University, Beijing, 100084 China; 4 University of Science and Technology of China, Hefei, 230026 China

## Abstract

Computed Tomography (CT) is a powerful method for non-destructive testing (NDT) and metrology awakes with expanding application fields. To improve the spatial resolution of high energy CT, a micro-spot gamma-ray source based on bremsstrahlung from a laser wakefield accelerator was developed. A high energy CT using the source was performed, which shows that the resolution of reconstruction can reach 100 μm at 10% contrast. Our proof-of-principle demonstration indicates that laser driven micro-spot gamma-ray sources provide a prospective way to increase the spatial resolution and toward to high energy micro CT. Due to the advantage in spatial resolution, laser based high energy CT represents a large potential for many NDT applications.

## Introduction

X-ray computed tomography (CT) has progressed significantly since its introduction in 1972^1^. As one of the most important non-destructive testing (NDT) method, CT has been widely used in medical radiology, industry, manufacturing, security and scientific researches.

CT is a three-dimensional (3D) X-ray imaging method that involves many projection images at different view angles around an axis through an object. Then, a tomographic image of a slice of the object can be obtained by using a reconstruction algorithm. High-energy CT using photons with energies greater than 1 MeV is often required for inspection of large and heavy manufactured items. In industrial applications, CT has been used to detect flaws and to determine the internal geometry of complex parts^2^, and it has become an important method to ensure quality control of individual components and their assembly in additive manufacturing^3^. Measurement precision is very important to those applications, however, high energy CT is still limited to sub-millimeter spatial resolution.

The CT spatial resolution is mainly determined by the spot size of the radiation source^4^. Micro-computed tomography, defined as spatial resolution less than 100 μm, has been achieved for low energy CT by the development of micro-spot X-ray machine^5^. High energy CT needs a bremsstrahlung source which is produced by MeV or higher electron beams from an electron linear accelerator^6,7^. The bremsstrahlung source usually has a spot size of 1–2 mm because of the focal spot size of the electron beam. Thus, the beam size is the limiting factor for the spatial resolution.

With the rapid development of intense laser technology, laser wakefield acceleration^8^ (LWFA) has become a new potential way to generate high-energy electron beams with excellent beam quality. The nonlinear behavior of the plasma wave allows to generate energetic and low-emanative electron beams in only few millimeters long underdense plasma^9^, which demonstrates the performance of laser accelerators as compact next-generation sources of energetic electrons and radiations^10,11^. Nowadays, high-quality electron beams can be reproduced steadily with peak energies from several hundreds of MeV to several GeV^12–16^. Because of the strong fields both in accelerating and focusing, that are thousands of times larger than those achievable in conventional accelerators, the transverse and longitudinal sizes of the electron bunch are extremely small (few μm), and comparable to the laser focal spot size (~10 μm) and pulse duration (~10 fs).

Many types of radiation can be obtained from this new accelerator^17^, which enable unprecedented potential in many applications. For example, betatron radiation from a laser plasma accelerator is an excellent X-ray source with an energy of several tens of keV, and with high spatial and temporal resolution. In recent years, the betatron sources have been developed with sufficient stability. Applications in low-energy CT for biomedicine have been demonstrated^18–21^, indicating a bright prospect for laser-based high-resolution CT technology.

By means of electron beams from LWFA, high-energy micro-spot gamma-ray sources can be also produced from bremsstrahlung. Pioneering work was performed by Glinec *et al*. in 2005^22^, an electron beam generated from LWFA had a beam divergence of about 17mrad (full-width half-maximum, FWHM) and a Maxwellian distribution with temperature of 40 MeV. A gamma-ray source with a spot size of 450 μm was generated with a 2.5 mm-thick tantalum converter. In 2011, Ben-Ismaïl *et al*.^23^ reduced the spot size to about 30 μm with a 1 mm-thick tantalum target by optimizing the electron beam divergence to about 3 mrad. To improve the photon yield, in 2016, Döpp *et al*.^24^ used argon or nitrogen gas in the LWFA, which supported a maximum charge of almost 1 nC per shot with a 1.1 J laser pulse. The bremsstrahlung photons were expected to have of ~10^−4^ J per shot, while the spot size was below 100 μm. Since 2012, we started our research on this subject^25^. In 2016, the properties and conditions of micro-spot gamma-ray source were detailed investigated^26^ to have a spot size is also as small as 40 μm by using a 5mrad electron beam. Two-dimensional (2D) radiography demonstrations with this gamma-ray source have high spatial resolution and penetration for high-area density objects.

With a spot size almost 20 times smaller than that of conventional gamma-ray sources, this method provides a breakthrough in resolution for high-energy CT. In this article, an improvement in the spatial resolution for high-energy CT is experimentally demonstrate by using a micro-spot gamma-ray source from a laser wakefield accelerator. Following our experiment result, intense laser provides a potential way to build a high-energy micro-CT system for NDT of complex and dense objects with a resolution less than 100 µm.

## Results

**Figure 1:**
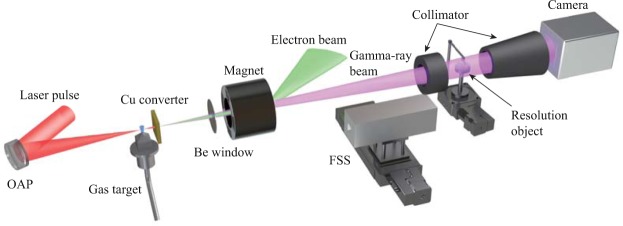
Experiment arrangement. A laser pulse focused by a F/6 off-axis parabola on a 0.7 mm gas jet with 1 mm height to generate low emittance electron beams with energies in the tens of MeV. Gamma-rays were produced by bremsstrahlung when the electron beams passed through a solid convertor. The gamma-ray beam then passed through a 300 µm-thick beryllium vacuum window before irradiating a test object. A 0.6 T magnet was placed after the vacuum window to deflect the residual electrons and to restrain secondary radiation background generated at the object. The point projection images were recorded by a high-energy X-ray camera which consisted of a CsI scintillator and a fiber-coupled CCD camera^47^. Two collimators were placed before and after the object to reduce the scattering gamma-ray photons. The filter stack spectrometer was used to measure the gamma-ray spectra and photon yields over the energy range of 0.01 MeV to 10 MeV.

In the experiment, the conditions of the micro-spot gamma-ray generation were chosen according to our previous research^26^. A 0.8 J, 28 fs laser pulse was focused on a gas jet with an outlet diameter of 0.7 mm to generate low emittance electron beams with energies of tens of MeV (see Fig. 1 and Methods). Gamma-ray photons were produced by bremsstrahlung when the electron beams passed through a solid convertor. The spot size was reduced with decreasing convertor thickness, and provided higher spatial resolution, however, the source intensity was also reduced. To balance the photon yield and spot size, a 0.5 mm-thick copper convertor was used to supports a 100 µm spot size^26^. A filter stack spectrometer (FSS)^27,28^ could be inserted into the gamma-ray beam before the object to measure the gamma-ray spectra and photon yields.

**Figure 2:**
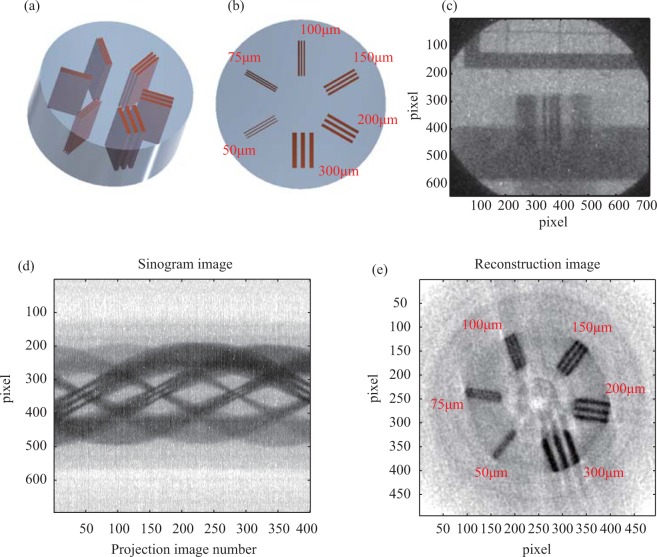
Spatial resolution object and CT imaging. (**a**) The CAD model of the spatial object, where copper plates were inlaid in a plastic cylinder. (**b**) Cross section image of the object. The resolution structures varied over 0.3–10LP/mm. (**c**) A projection image of the spatial object after two collimators. The object was placed on an aluminum holder, connected to the rotation stage. (**d**) A sinogram image of a slice of the object generated from 400 projection images at 0.5° intervals. (**e**) The reconstructed tomography image from (**d**) using a filtered back projection (FBP) algorithm.

A special resolution object was placed 100 cm away from the gamma-ray source. The projection images were rerecorded by a high-energy X-ray camera 30 cm away from the object. The geometry resulted in a magnification of 1.3 that was optimized before the experiment and predicts a satisfactory spatial resolution of near the 85 μm^29^. Following the CT standard^30,31^, an object, a perspex cylinder with some copper plates embedded inside to form periodic structures was made to test the spatial resolution. As shown in Fig. 2(a,b), each group of structures had three periods with the line width from 300 µm to 50 µm, and pointed to the symmetric axis. The whole object had a diameter of 12 mm and height of 5 mm and was located on a rotation stage. A typical projection image is shown in Fig. 2(c). A cross-line marker made of 300 µm molybdenum (Mo) wire was placed above the object and was fixed independent of the rotation stage. It was used to inspect the source positron drift from the shot-to-shot fluctuations, and to register the projection images.

A total of 400 projection images were obtained from 1° to 200°, and each image was the accumulation of three laser shots. Between each images, a 0.5 degree rotation of the object is applied. A sinogram of one slice of the object, shown in Fig. 2(d), is collected a same row from 400 projection images. After removing the noise via image processing, the image intensities were logarithmically converted into linear attenuation coefficient *μ*. In post-processing, all projection images were registered according to the positrons of the stationary marker. Then the reconstructed image was obtained by using a filtered back projection (FBP) algorithm. The reconstruction matrix was 500 × 500, and the corresponding voxel size was 28 × 28 × 28 µm^3^. The Fig. 2(e) shows the reconstructed slice image in which the 100 µm structure can be resolved.

**Figure 3:**
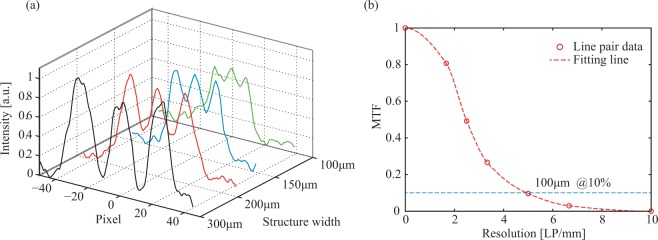
CT resolution. (**a**) The cross sections of each periodic structure (300–100 μm). (**b**) Dots indicate the image contrast from each cross section curve. A fitting line indicates that the system spatial resolution is 100 μm with a 10% contrast standard.

To assess spatial resolution, a modulation transfer function (MTF) curve was obtained from the cross sections of the periodic structures^30^, as shown in Fig. 3. According to the 10% evaluation standard^31^, the full system spatial resolution reaches 100 μm, and has enter the range of micro CT (resolution ≤ 100 μm).

## Discussion

In our experiment, the 20 s time interval between laser shots was determined by the gas loading, vacuum pump ability and the data transport speed. The whole CT test took almost 7 hours, which implies high system stability. From the location and intensity of the fiducial in projection images, the gamma-ray source had a position distribution within an 86 μm radial and a 10% brightness fluctuation about the normal distribution 1*σ*. The source position fluctuation was attributed to laser angle drift, electron pointing instability and environmental vibration. By optimization of the laser performance, the target design and the system structure, the stability could be greatly improved. For example, the source dispersion could be decreased by one order of magnitude by controlling the laser contrast^32^. The integral source size during the image accumulation could be also reduced to the further improve spatial resolution.

The image quality and the spatial resolution of reconstruction are also affected by reconstruction method. In our demonstration, the traditional FBP algorithm created many artefacts in the reconstruction image. Many advanced methods have been developed that provide more powerful noise processing, artefact handling, reconstruction efficiency and quality^33,34^. Thus, the image quality of laser based high-energy tomography can be further improved by using these advanced reconstruction methods, as shown in refs^21,22^.

**Figure 4:**
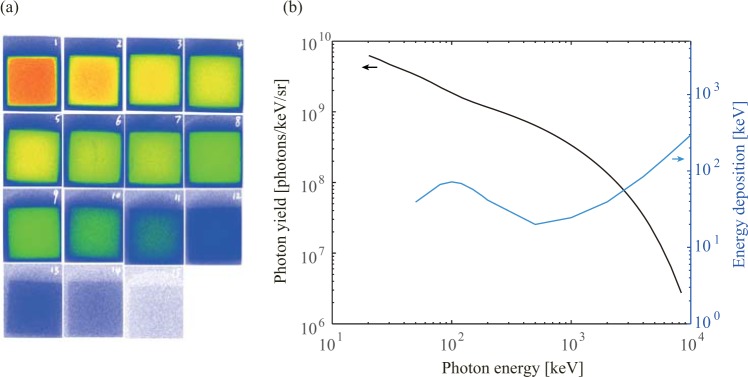
Gamma-ray spectrum from FFS measurements. (**a**) Raw images from the filter stack. Fifteen filters with different materials and thicknesses were used to measure the gamma-ray energy spectra over 0.01–10 MeV^28^. (**b**) Gamma-ray energy spectrum reconstructed from the transmission signals of the FSS. The absolute photon number can be obtained from image plate calibration data. Also plotted curve shows the photon energy deposited in the CsI scintillator used in the high-energy X-ray camera^47^.

The photon spectra and photon yields were obtained from the results of FSS, as shown in Fig. 4. The radiation dose can be calculated from these results, and it was also measured with an ionization chamber detector. Both methods yielded a dose estimation of 30 µGy/shot@1 m. The dose per minute was still a big difference relative to a conventional industrial CT system, and was mainly limited by the 0.8 J laser pulse energy and 0.05 Hz system repetition. High beam charge acceleration could generate an electron bunch with the charge of nC per J laser energy^24,35^, which would increase the radiation dose of single laser pulse by an order of magnitude. With the further development in laser technology, high peak power and high repetition rate laser systems will become more industrial and commercial^36^. In the future, high flux gamma-ray sources will greatly improve the dose rate and testing efficiency, which could be comparable to conventional systems that complete a CT test in several minutes.

The CsI based high energy X-ray camera was not ideal for high energy radiography. In industrial application, to achieve a high quality image, the use of standard 2D detectors is limited because of the dominating absorption effect of Compton scattering at high energies, which will bring severe cross talk between the pixels. Especially for CT reconstruction purposes, these effects have to be considered on the development path from classical 2D radioscopy to 3D imaging. Most high energy CT applications are therefore adopting line detectors with a slit collimator to shield the scattered radiation and increase the imaging quality. Commercial line detectors usually have high efficiency scintillator of 10 mm CdWO_4_ that giving an absorption of 25% over 1–10 MeV, and still keeping 100 μm spatial resolution. As the prediction of our optimization (see Methods and ref.^29^), even for 100 g/cm^2^ area density object, a spatial resolution can be improved to near 70 μm with a 100 μm source, a 100 μm unit size line detector, and 1.5x magnification. Due to the time consumption when using a linear detector in 3D reconstruction, new generation panel area detectors, such as amorphous silicon flat panel area detector^37^, multi-pixel CdTe detector^38^ are continuously improved for high quality industrial radiography. These detectors offer very high sensitivity, resolution, and bit depth, resulting in 2D images with clarity and contrast, supports fast 3D non-destructive testing.

In summary, a proof-of-principle demonstration of high-resolution high-energy CT was presented by using a micro-spot gamma-ray source driven by a compact laser plasma accelerator. The reduced spot size of the laser driven source improved the spatial resolution of reconstruction to 100 μm. The resolution was mainly limited by the source position drift, which could be further improved by controlling the laser system, the plasma accelerator and the target performances. A high-repetition-rate intense laser could increase the radiation dose to the same level as a conventional CT system. Our demonstration shows that the laser-based gamma-ray source provides a bright future for high energy micro CT with resolution of tens of microns which has considerable potential in non-destructive testing for many applications.

## Methods

### Laser wakefield accelerator

The experiment was performed with a commercial femtosecond laser system (Amplitude Technology) at the Science and Technology on Plasma Physics Laboratory. With an off axis parabolic mirror with focal-length of 333 mm, the 55 mm diameter laser beam was focused to a 6.9 μm spot (radial at 1/e^2^), which contained 58% of the laser energy. The pulse duration could be compressed to as short as 24 fs, giving a peak intensity of about 2.9 × 10^19^ W/cm^2^ at the target point for an energy of 0.8 J. A 0.7 mm-diameter gas jet was used to generate electron beams with energies of tens of MeV. Pure nitrogen was used to generate much higher plasma density and to increase the total charge by ionized injection^39,40^. The density profile was measured with an interferometer, which represents a near Gaussian distribution with a 1 mm width at FWHM. The molecular density could be varied over 1.8 × 10^18^ cm^−3^ to 2.86 × 10^19^ cm^−3^ for a back pressure of 100–2000 kPa. According to our previous optimization, with the plasma density of 8.3 × 10^19^ cm^−3^, the beam divergence was controlled to about 5 mrad (Supplementary Fig. S1), with an electron energy near 40 MeV (Supplementary Fig. S2). The total charge of the electron beam was between 100 pC and 130 pC.

### High energy gamma-ray properties

The high energy gamma-rays were generated from the bremsstrahlung of the energetic electron beams propagating in high Z materials. The photon energy spectra always obeyed the quasi-Maxwellian or exponential distribution, and was almost independent to the electron spectra. Spectra for gamma-rays higher than 50 keV could not be measured by dispersion methods. Instead, a filter stack technology was used with fourteen filters of different materials and thicknesses that covered the energy range 0.01–10 MeV. The gamma-rays deposited its energy in image plates (IP) between the filters and generated signals with different intensities. The gamma-ray spectrum was obtained by solving the signal intensities on each IP, the transmission function of each filters and the energy response function of IP^27,28,41^. With the calibration data of the IP response^42–44^, the photon yield could be also obtained^45^. According to the absolute gamma-ray spectrum, the radiation dose could be obtained from using flux-to-dose conversion factors^46^. Before the CT demonstration, a projection image of a 2D resolution test object, used in our previous experiment^26^, was obtained to check the spatial resolution of the imaging system. (Supplementary Fig. S3).

### CT geometry

In CT applications, the spatial resolution is mainly determined by the X-ray spot size and detector aperture, but also largely affected by geometry. Thus an optimal designing was performed via the resolution formula and simulations. In imaging theory, spatial resolution can be evaluated by the equivalent beam width^30,31^

$$BW=\frac{\sqrt{{d}^{2}+{(a(M-1))}^{2}}}{M}$$

Here, *a* is spot size, *d* is detector aperture and *M* is geometry magnification. According to the resolution evaluation, with a 150 μm laser-driven spot and a 100 μm-aperture commercial detector, the magnification was optimized to 1.3 to 1.5, giving a resolution of ~85 μm. Concerning the X-ray beam divergence angle, a 100 cm distance between the source and the object provided a satisfactory view field. The geometry was also optimized with simulations, discussed in detail elsewhere^29^. The simulation results supported the design and provided satisfied resolution even with some source position drift, spot size fluctuation and Poisson noise.

### CT reconstruction

The gamma-ray photons were damped according to the material thickness of the object. The intensity *I* in a projection image follows the Beer-Lambert law, *I*/*I*_0_ = exp(−*μt*), where *I* is the intensity attenuated by object, *I*_0_ is the intensity without object, *μ* is the attenuation coefficient, *t* is path length in object. In CT measurement, the aim is to obtain the distribution of linear attenuation coefficient *μ* in a slice. The intensity image should be convert into the integration of *μ* along the gamma-ray path. In the demonstration, the object diameter was much less than the distance of the source to the object; hence, the measurement could be treated as parallel beam projection. Before the reconstruction, all images were registered by the positrons of the cross line marker. A classical FBP algorithm was used for reconstruction. The object function in a slice could be expressed as *f*(*x*,*y*), the projection of this slice could be expressed by the rotation angle *θ* and the distance of rotation center to a beam line *u* as

$$p(\theta ,u)=\int {\rm{dxdy}}f(x,y)\delta (x\,\cos \,\theta +y\,\sin \,\theta -u)$$

where *σ* is Dirac delta function. This expression is the famous 2D Radon transformation.

We first calculate the Fourier transform of *p*(*θ*, *u*)

$$P(\theta ,\upsilon )=\int {\rm{d}}up(\theta ,u){e}^{-2\pi i\upsilon u}=\int {\rm{d}}x{\rm{d}}yf(x,y){{\rm{e}}}^{-2\pi i\upsilon (x\cos \theta +y\sin \theta )}.$$

Then, the object function can be obtained by 2D inverse Fourier transform,

$$f(x,y)={\int }_{0}^{\pi }{\rm{d}}\theta {\int }_{-\infty }^{\infty }d\upsilon |\upsilon |P(\theta ,\upsilon ){{\rm{e}}}^{2\pi i\upsilon u}|{}_{u=x\cos \theta +y\sin \theta }$$

## Electronic supplementary material

Electron beam properties and DR resolution test
