# Supplementary material for: Towards high-energy, high-resolution computed tomography via a laser driven micro-spot gamma-ray source
Source: Sci Rep. 2018 Oct 26;8:15888. doi: 10.1038/s41598-018-33844-7 (PMC6203838; doi:10.1038/s41598-018-33844-7)

Towards high-energy, high-resolution computed tomography via a laser driven micro-spot gamma-ray source

Y. C. Wu, B. Zhu, G. Li, X. H. Zhang, M. H. Yu, K. G. Dong, T. K. Zhang, Y. Yang, B. Bi,

J. Yang, Y. H. Yan, F. Tan, W. Fan, F. Lu, S. Y. Wang, Z. Q. Zhao, W. M. Zhou,

L. F. Cao & Y. Q. Gu

Supplemental material

**Supplementary Figure S1.** Typical electron beam profile measured by a DRZ screen which placed outside the vacuum chamber and has a distance of 297mm from the laser focal spot. At the optimal working point, the beam divergence was about 5mrad.


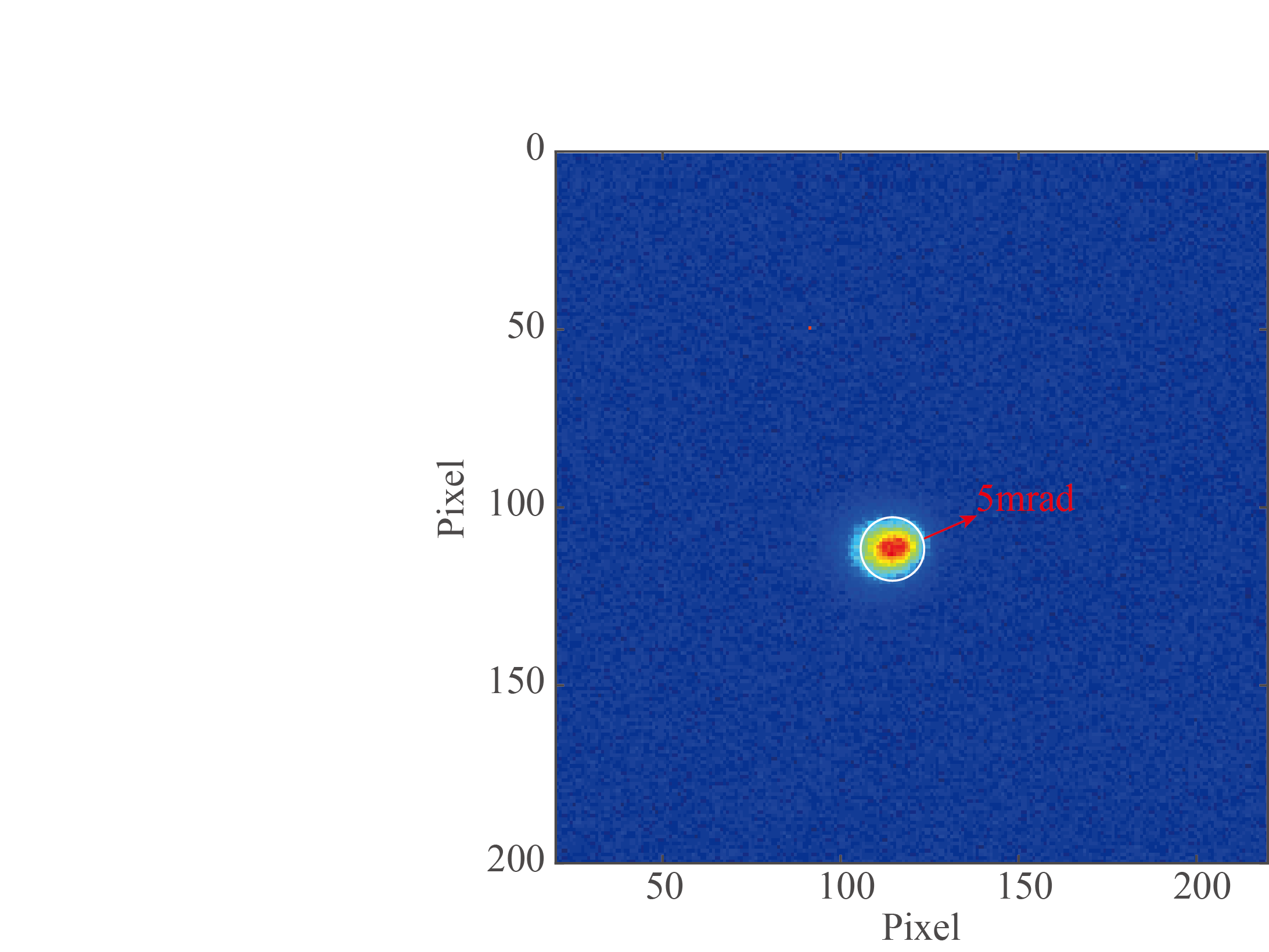


**Supplementary Figure S2**. Typical electron spectrum measured with a 0.6T magnetic spectrometer placed after the Be vacuum window. Due to the ionized injection, the electron beam had a wide spectrum with energy near 40MeV. The gap in the spectrum image occurred because the electron beam was recorded by two DRZ screens.


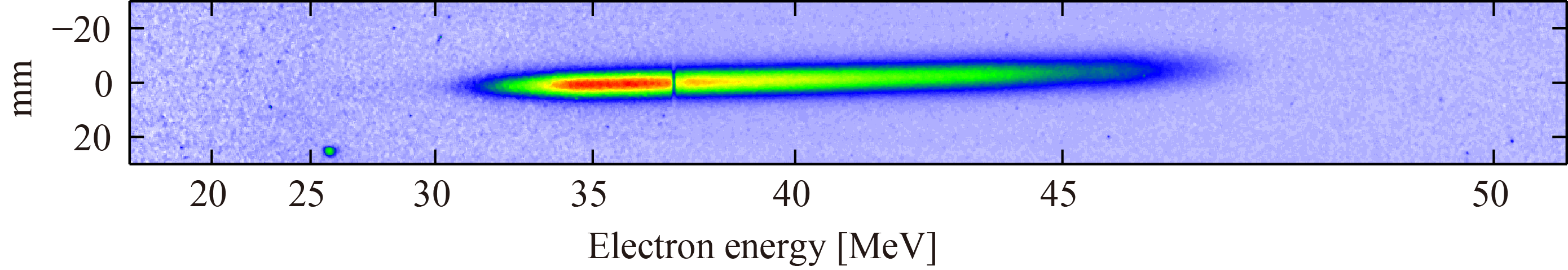


**Supplementary Figure S3.** Projection image of the spatial test object made of a 10mm-thick stainless steel plate with different periodic structures. The line widths of the periodic structures varied from 0.2mm to 0.8mm. The imaging arrangement was same as that for the CT demonstration.


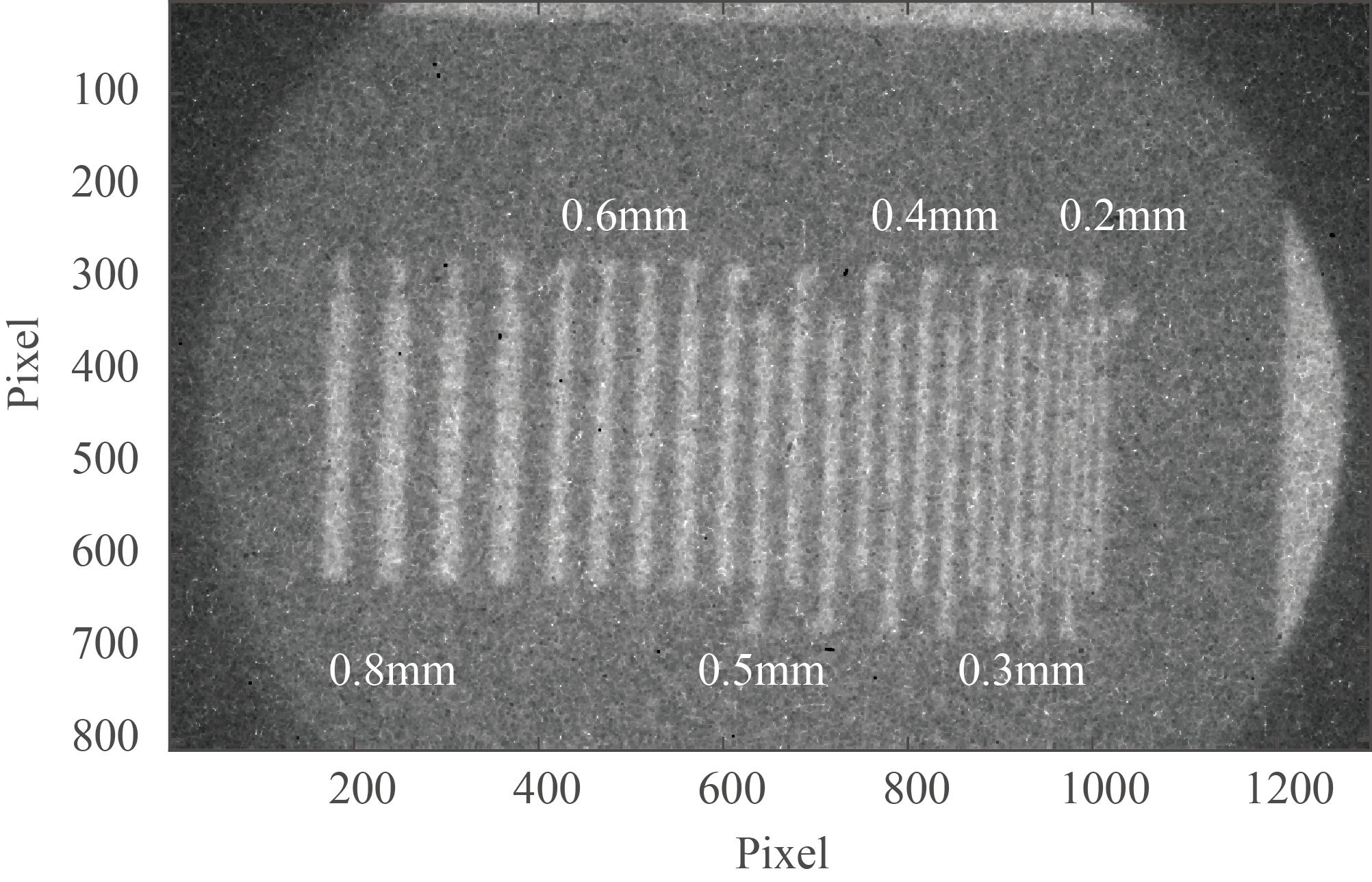

Supplement: Supplementary file 1 — Electron beam properties and DR resolution test [file 41598_2018_33844_MOESM1_ESM.docx]
